# Phase 1b trial of proteasome inhibitor carfilzomib with irinotecan in lung cancer and other irinotecan-sensitive malignancies that have progressed on prior therapy (Onyx IST reference number: CAR-IST-553)

**DOI:** 10.1007/s10637-017-0441-4

**Published:** 2017-02-16

**Authors:** Susanne M. Arnold, Kari Chansky, Markos Leggas, Michael A. Thompson, John L. Villano, John Hamm, Rachel E. Sanborn, Glen J. Weiss, Gurkamal Chatta, Maria Q. Baggstrom

**Affiliations:** 1 University of Kentucky Markey Cancer Center, 800 Rose Street CC445, Lexington, KY 40536 USA; 2 University of Kentucky Department of Internal Medicine, Lexington, KY USA; 3 Cancer Research and Biostatistics (CRAB), Seattle, WA USA; 4 University of Kentucky Department of Pharmaceutical Sciences, Lexington, KY USA; 5 Aurora Research Institute, Milwaukee, WI USA; 6 University of Kentucky Department of Medicine, Lexington, KY USA; 7 Norton Cancer Institute, Louisville, KY 40202 USA; 8 Providence Cancer Center, Portland, OR USA; 9 Cancer Treatment Centers of America At Western Regional Medical Center, Goodyear, AZ USA; 10 Roswell Park Cancer Institute, Buffalo, NY USA; 11 Washington University School of Medicine Department of Internal Medicine, St. Louis, MO USA

**Keywords:** Lung cancer, Carfilzomib, Phase I, Irinotecan

## Abstract

*Introduction* Proteasome inhibition is an established therapy for many malignancies. Carfilzomib, a novel proteasome inhibitor, was combined with irinotecan to provide a synergistic approach in relapsed, irinotecan-sensitive cancers. *Materials and Methods* Patients with relapsed irinotecan-sensitive cancers received carfilzomib (Day 1, 2, 8, 9, 15, and 16) at three dose levels (20/27 mg/m2, 20/36 mg/m2 and 20/45 mg/m2/day) in combination with irinotecan (Days 1, 8 and 15) at 125 mg/m2/day. Key eligibility criteria included measurable disease, a Zubrod PS of 0 or 1, and acceptable organ function. Patients with stable asymptomatic brain metastases were eligible. Dose escalation utilized a standard 3 + 3 design. *Results* Overall, 16 patients were enrolled to three dose levels, with four patients replaced. Three patients experienced dose limiting toxicity (DLT) and the maximum tolerated dose (MTD) was exceeded in Cohort 3. The RP2 dose was carfilzomib 20/36 mg/m^2^ (given on Days 1, 2, 8, 9, 15, and 16) and irinotecan 125 mg/m2 (Days 1, 8 and 15). Common Grade (Gr) 3 and 4 toxicities included fatigue (19%), thrombocytopenia (19%), and diarrhea (13%). *Conclusions* Irinotecan and carfilzomib were well tolerated, with common toxicities of fatigue, thrombocytopenia and neutropenic fever. Objective clinical response was 19% (one confirmed partial response (PR) in small cell lung cancer (SCLC) and two unconfirmed); stable disease (SD) was 6% for a disease control rate (DCR) of 25%. The recommended phase II dose was carfilzomib 20/36 mg/m^2^ and irinotecan125 mg/m2. The phase II evaluation is ongoing in relapsed small cell lung cancer.

## Introduction

Small cell lung cancer (SCLC) is well recognized for its initial sensitivity to chemotherapy, but ultimate recurrence and resistance to subsequent lines of therapy. The overall prognosis remains poor, with a two-year overall survival of less than 5% and a median survival of approximately nine to 11 months [1], attributable to the lack of effective salvage regimens for this disease. Currently, Food and Drug Adminstration (FDA)-approved second-line therapies provide minimal extension of life. We developed a novel combination therapy using irinotecan and a proteasome inhibitor, carfilzomib, and report the initial safety profile of this combination.

The proteasome is a multicatalytic proteinase complex that first ubiquinates and then degrades a variety of protein substrates within normal and transformed cells. Carfilzomib is a tetrapeptide ketoepoxide-based proteasome inhibitor specific for the chymotrypsin-like active site of the 20S proteasome. Proteasome inhibition by carfilzomib interrupts cellular pathways integral to the survival of small cell lung cancer [2–4], namely the dysregulated apoptotic pathway involving activated nuclear factor-kB (NF-kB) [5]. NF-kB activates the transcription of anti-apoptotic and proliferation genes, mediating tumor cell survival in response to cytotoxic stress and resulting in chemoresistance, a common problem in SCLC. Carfilzomib prevents proteasomal degradation of IkB, the inhibitor of NF-kB, and also modulates levels of the anti-apoptotic gene Bcl-2 and the tumor suppressor p53. Overexpression of Bcl-2, a key mediator of resistance to apoptosis following chemotherapy, is common in SCLC [4], and low levels of bcl-2 and b1-integrin are associated with improved survival in SCLC [6]. Topoisomerase-1 is also overexpressed in SCLC [5] and is thought to cause apoptosis via mechanisms other than NF-kB, adding to the potential synergy of these compounds.

The results of in vitro and in vivo studies demonstrated the importance of prolonged proteasome inhibition to anti-tumor efficacy [7, 8] and the optimal dosing schedule of carfilzomib as twice-weekly (Day 1 and 2) in a variety of hematologic and solid tumor types. However, other schedules including once weekly dosing are being explored in the clinic (NCT02335983).

Single-agent phase I studies of carfilzomib in solid tumors revealed responses in SCLC, which led to the conception of the present phase I, study in combination with irinotecan [9]. Irinotecan was chosen for its well-established activity in SCLC as a single agent [10–12] and in combination with platinum [13, 14]. Inactivation of proteasome function allows for increased apoptosis and potential for enhanced antitumor effects through enhanced apoptosis and non-cross reactive mechanisms, and interference with Topo-I degradation. Based on the hypothesis that carfilzomib would enhance the anti-tumor efficacy of irinotecan in relapsed irinotecan-sensitive cancers, we undertook the present phase I study to determine the maximum tolerated dose (MTD) of this combination.

## Material and methods

The primary objective was to determine MTD of carfilzomib (Day 1, 2, 8, 9, 15, and 16) in combination with irinotecan (Days 1, 8 and 15) in subjects with relapsed irinotecan-sensitive cancers including small and non-small cell lung cancer (NSCLC). Secondary objectives included response rate (RR), safety/tolerability, and biomarker endpoints of carfilzomib proteasome chymotrypsin-like activity in Is this peripheral blood mononuclear cell (PBMC) (LMP7 and b5 activity).

All subjects were informed of the investigational nature of this study and signed Institutional Review Board (IRB)-approved informed consent documents in accordance with institutional and federal guidelines. Subjects were at least 18 years of age, with Zubrod performance status 0 or 1, and histologically or cytologically-confirmed recurrent or progressive irinotecan-sensitive cancer with no curative therapeutic options. Subjects had measurable disease by Response Evaluation Criteria In Solid Tumors (RECIST*)* criteria [15], brain metastasis treated and asymptomatic if present, no other therapy within: 14 days (chemotherapy), 21 days (radiation), or 28 days (surgery) prior to enrollment and recovery from all associated toxicities of these therapies. Subjects had normal bone marrow, renal and hepatic function assessed within 14 days prior to enrollment. Pregnant or nursing females were excluded and all subjects agreed to use an effective contraceptive method. Exclusion criteria included: prior use of irinotecan or carfilzomib; leptomeningeal metastases; progression during or within one month of completion of first-line platinum-based chemotherapy; active infection, including human immunodeficiency virus (HIV) or hepatitis A, B or C; unstable angina or myocardial infarction within preceding four months, New York Heart Association Class III or IV heart failure, uncontrolled angina, significant conduction system abnormalities; uncontrolled hypertension or diabetes; evidence of moderate or severe pulmonary hypertension; significant neuropathy (Grades [Gr] 3–4, or Gr 2 with pain); known allergy to Captisol®; other clinically active cancer and or history of prior malignancy within the past three years (exceptions: basal cell or squamous skin cancer, thyroid cancer; carcinoma in situ of the cervix or breast; prostate cancer of Gleason Gr 6 or less with stable prostate-specific antigen levels; or other cancer considered cured by surgical resection).

Dose escalation is listed in Table 1. Per carfilzomib investigator brochure and safety instructions, “stepped-up” dosing was required for all subjects on Cycle 1 Day 1 20 mg/m2, with all subsequent doses as assigned by dose escalation schema. Doses were calculated using the subject’s actual body surface area (BSA), however, subjects with a BSA > 2.2 m2 received a capped dose based on a 2.2 m2 BSA. Dose adjustments were made for greater than 10% change in body weight. Dexamethasone premedication (8 mg) and intravenous (i.v.) prehydration (250 to 500 mL of normal saline) was given prior to all doses of carfilzomib during Cycle 1 and at investigator discretion thereafter. Carfilzomib was supplied as a lyophilized parenteral product in single-use vials and reconstituted with sterile water for injection to a final carfilzomib concentration of 2.0 mg/mL. Commercially available irinotecan was diluted with D5W or normal saline to a final concentration of 0.12 to 2.8 mg/ml and administered via i.v. over 90 min on dDays 1, 8, and 15. Carfilzomib was administered via i.v. over 30 min on Days 1, 2, 8, 9, 15, and 16, after irinotecan. Additional cycles of therapy could be administered provided the subject had: absolute neutrophil count (ANC) ≥ 1500/μL, platelets ≥100,000/μL, resolution of all other Gr 2, 3 or 4 non-hematological toxicities and a creatinine ≤1.5 x IULN.

**Table 1:** Phase 1b dose escalation scheme

3 + 3 design	Doses	
	Carfilzomib*	Irinotecan
Cohort −2	20 mg/m2	75 mg/m2
Cohort −1	20 mg/m2	100 mg/m2
Cohort 1	20/27 mg/m2	125 mg/m2
Cohort 2	20/36 mg/m2	125 mg/m2
Cohort 3	20/45 mg/m2	125 mg/m2
Cohort 4	20/56 mg/m2	125 mg/m2
Cohort 5	20/70 mg/m2	125 mg/m2

Cycle 1 Day 1 and Day 2 doses are 20 mg/m2. All subsequent days as specified, i.e. 20/27 mg/m2 means Cycle 1 Day 1 and Day 2 doses are 20 mg/m2 and all other days are 27 mg/m2

Subjects were evaluated for toxicity according to the Common Terminology Criteria for Adverse Events (CTCAE) version 4.0. A dose limiting toxicity (DLT) was defined as any of the following treatment emergent toxicities with attribution (possibly, probably or definitely related) to one or more of the study drugs that occur during Cycle 1: ≥ Gr 2 neuropathy with pain; any Gr 3 or 4 adverse event (excluding Gr 3 fatigue, nausea, vomiting, diarrhea lasting <7 days); Gr 3 or 4 nausea, vomiting, or diarrhea lasting >7 days despite maximal antiemetic/antidiarrheal therapy; ≥ Gr 3 non-hematologic laboratory findings if determined to be clinically significant, Gr 4 neutropenia lasting for ≥7 days; febrile neutropenia; Gr 4 thrombocytopenia lasting ≥7 days; Gr 3 or 4 thrombocytopenia with bleeding; or any Gr 5 toxicity. Patients were evaluable for DLT if they receive the assigned doses and schedule of chemotherapy throughout Cycle 1 or develop a DLT. If a patient did not develop a DLT, but did not complete Cycle 1 for any reason, they were considered not evaluable for DLT and replaced. Dose reductions/adjustments were defined in the protocol and no dose re-escalation was allowed. Criteria for removal from protocol included: completion of six cycles of chemotherapy, progressive disease (PD), symptomatic deterioration resulting in unacceptable toxicity, treatment delays greater than three weeks, or removal at the discretion of the treating physician or withdrawal by patient.

Serial blood samples were collected on Day 1 pre-irinotecan, and at ~90 min, ~2 h, ~5.5 h (relative to the start of irinotecan), and Day 2: pre-carfilzomib infusion. The dose dependent proteasome inhibition was determined in purified PBMC by measuring the chymotrypsin-like activity.

The primary endpoint for this phase I trial was determination of the MTD. The decision rules followed the modified Fibonnaci design (3 + 3). With this design, at least six patients are evaluated at the dose level chosen as the MTD. Assuming that a Cycle 1 DLT rate of 25% is acceptable, if the true rate of Cycle 1 DLT were 40%, and a dose level is rejected with two or more out of six experiencing DLT, this design has 77% power to reject the null hypothesis of an acceptable DLT rate.

The secondary endpoints of progression-free survival (PFS), response to treatment, and rates of adverse events were calculated based on all eligible subjects who received at least the first day of treatment. Survival was estimated using the Kaplan-Meier method. Responses were evaluated by RECIST version 1.1 approximately every six weeks. Objective responses were confirmed at least four weeks later. PFS is defined as the date of registration to the date of first documentation of progression by RECIST 1.1 criteria, or death due to any cause. Subjects last known to be alive and progression free are censored at the date of last contact. Due to the limited samples size in this study, analyses of secondary endpoints and correlative studies were considered to be exploratory in nature.

## Results

### Patient characteristics and treatment delivery

From October 30, 2013 to January 20, 2015, 22 patients were screened and 16 patients were enrolled and treated on this phase Ib trial after providing informed consent. Four patients had to be replaced because of Cycle 1 treatment could not be completed and a DLT was not observed. Baseline characteristics are listed in Table 2. The majority of enrolled subjects had lung cancer (two NSCLC, 13 SCLC). One subject had ovarian cancer. Fifteen patients (93%) were Caucasian, 50% were male.

**Table 2:** Patient characteristics for 16 eligible patients

Patient Characteristics	Cohort 1: 20/27 mg/m2 Carfilzomib, 125 mg/m2 Irinotecan		Cohort 2: 20/36 mg/m2 Carfilzomib, 125 mg/m2 Irinotecan		Cohort 3: 20/45 mg/m2 Carfilzomib, 125 mg/m2 Irinotecan		Overall	
Sex								
Female	3	(75%)	4	(44%)	1	(33%)	8	(50%)
Male	1	(25%)	5	(55%)	2	(66%)	8	(50%)
Performance Status								
0	0	0	3	(33%)	0	0	3	(18%)
1	4	(100%)	6	(66%)	3	(100%)	13	(81%)
Histology								
Adenocarcinoma (Lung)	1	(25%)	0	0	0	0	1	(6%)
Ovarian	0	0	1	(11%)	0	0	1	(6%)
SCLC	3	(75%)	7	(77%)	3	(100%)	13	(81%)
Mixed SC/NSCLC	0	0	1	(11%)	0	0	1	(6%)
Race								
Black	1	(25%)	0	0	0	0	1	(6%)
White	3	(75%)	9	(100%)	3	(100%)	15	(93%)

Overall, 16 patients received at least one day of chemotherapy. One subject completed all cycles of therapy, 12 subjects discontinued treatment prior to six cycles due to disease progression, two subjects refused further treatment for reasons other than adverse events, and one subject withdrew because adverse events related to treatment were intolerable to the patient. This patient refused to continue at a lower irinotecan dose. Overall, one subject completed four and one completed six cycles of therapy, two subjects completed two and three cycles of therapy, respectively, six completed one cycle of therapy and four subjects did not complete the first cycle of therapy.

One patient on Cohort 3 had a dose delay at Cycle 2 Day 1 due to thrombocytopenia (a DLT from Cycle 1). This patient was able to continue with treatment after delay and completed three cycles. A patient in Cohort 2 had the Cycle 2 dose reduced for both drugs due to Gr 1 (GI) gastrointestinal toxicities and fatigue, and continued on to complete four cycles. Another patient in Cohort 2 was required to hold Days 8 and 9 for creatinine clearance, successfully restarting on day 15. A patient on Cohort 3 was held for Cycle 1 Days 15 and 16 due to diarrhea (a DLT) and subsequently restarted with Cycle 2 after a delay and a reduction to dose level 2. This patient received four cycles of treatment.

### Safety

Three patients experienced DLTs with one in Cohort 2 (dehydration), and two in Cohort 3 (dehydration and diarrhea), exceeding the MTD in Cohort 3. With only one DLT in six patients in Cohort 2, the MTD was 20/36 mg/m^2^ of carfilzomib (given on Days 1, 2, 8, 9, 15, and 16) and 125 mg/m2 of irinotecan (Days 1, 8 and 15). The course of the study, one patient on Cohort 1 and three patients on Cohort 2 were not evaluable for DLT because they did not finish Cycle 1. All toxicities (with attribution ruled as possibly, likely, or definitely related to treatment) are listed in Table 3. Common Gr 3 and 4 toxicities included: fatigue (19%), thrombocytopenia (19%), and diarrhea (13%). At Gr 3, there was one report each of: hyponatremia, muscle weakness, hypocalcemia, dehydration, anemia, nausea, and vomiting. At Gr 4, there was one report each (6% of patients) of: decreased neutrophils, decreased lymphocytes, and decreased white blood cells.

**Table 3:** Adverse events over all treatment cohorts. Events possibly, likely, or definitely related to treatment are included

	Maximum Grade AEs with attribution Possible Probable or Definite				
All Eligible Patients	(N = 16)				
Adverse Event Description	1	2	3	4	5
Blood and lymphatic system disorders		1 (6%)	1 (6%)		
Anemia		1 (6%)	1 (6%)		
Cardiac disorders	1 (6%)				
Cardiac disorder-Other, specify	1 (6%)				
Gastrointestinal disorders	4 (25%)	5 (31%)	3 (19%)		
Abdominal pain		1 (6%)			
Constipation	1 (6%)				
Diarrhea	2 (13%)	4 (25%)	2 (13%)		
Nausea	4 (25%)	2 (13%)	1 (6%)		
Vomiting	2 (13%)		1 (6%)		
GI disorders-Other, specify	2 (13%)				
General disorders and administration site conditions	2 (13%)	3 (19%)	3 (19%)		
Edema limbs	1 (6%)				
Fatigue	3 (19%)	2 (13%)	3 (19%)		
Infusion related reaction		1 (6%)			
Infections and infestations		2 (13%)			
Urinary tract infection		2 (13%)			
Vaginal infection		1 (6%)			
Injury, poisoning and procedural complications	1 (6%)				
Fall	1 (6%)				
Investigations	1 (6%)	2 (13%)	2 (13%)	2 (13%)	
ALT increased	1 (6%)				
AST increased	1 (6%)				
Alkaline phosphatase increased	1 (6%)				
Creatinine increased		1 (6%)			
Lymphocyte count decreased				1 (6%)	
Neutrophil count decreased		1 (6%)	1 (6%)	1 (6%)	
Platelet count decreased		1 (6%)	3 (19%)		
Weight loss	1 (6%)				
White blood cell decreased		2 (13%)		1 (6%)	
Investigations-Other, specify	1 (6%)				
Metabolism and nutrition disorders	1 (6%)	2 (13%)	3 (19%)		
Anorexia	1 (6%)	1 (6%)			
Dehydration	1 (6%)	1 (6%)	1 (6%)		
Hypoalbuminemia		1 (6%)			
Hypocalcemia			1 (6%)		
Hypokalemia		1 (6%)			
Hypomagnesemia		1 (6%)			
Hyponatremia	1 (6%)		1 (6%)		
Musculoskeletal and connective tissue disorders			1 (6%)		
Generalized muscle weakness			1 (6%)		
Nervous system disorders	3 (19%)				
Dizziness	1 (6%)				
Peripheral sensory neuropathy	1 (6%)				
Tremor	1 (6%)				
Psychiatric disorders	2 (13%)				
Confusion	1 (6%)				
Insomnia	1 (6%)				
Renal and urinary disorders	1 (6%)	1 (6%)			
Urinary retention		1 (6%)			
Renal/urinary disorders-Other	1 (6%)				
Respiratory, thoracic and mediastinal disorders		1 (6%)			
Atelectasis		1 (6%)			
Productive cough	1 (6%)				
Skin and subcutaneous tissue disorders	2 (13%)				
Alopecia	1 (6%)				
Rash maculo-papular	1 (6%)				
Skin/subq tissue ds-Other	1 (6%)				
Vascular disorders	1 (6%)	1 (6%)			
Hot flashes	1 (6%)				
Hypotension		1 (6%)			
Maximum Grade Any Toxicity	3 (19%)	4 (25%)	6 (38%)	2 (13%)	0

### Pharmacodynamics

**Fig. 1:**
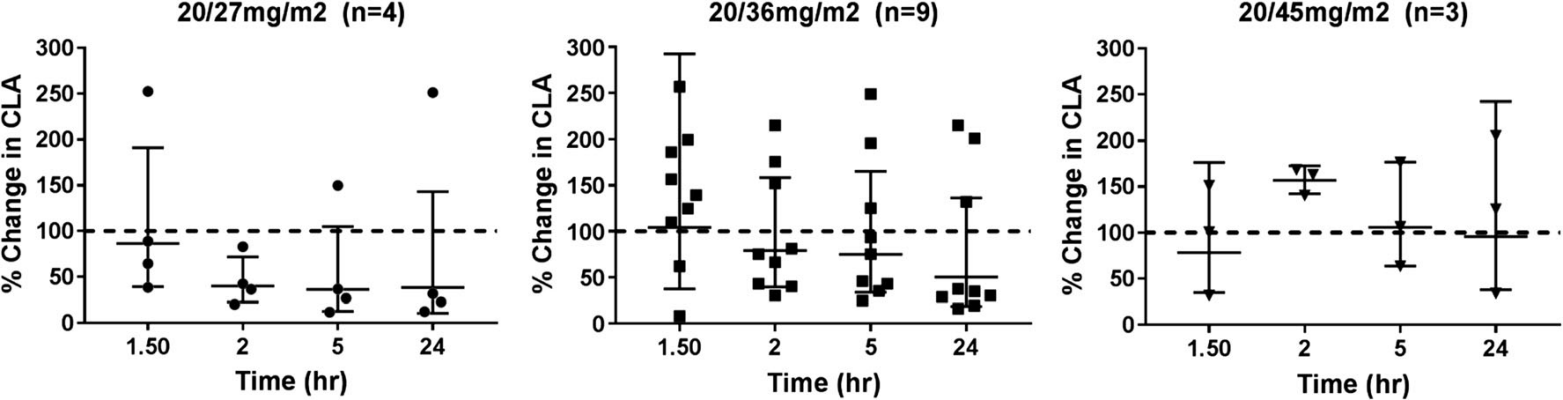
Time dependent change, relative to pretreatment, of the chemotrypsin-like activity (CLA) in isolated PBMC from patients receiving 20/27, 20/36, and 2045 mg/m^2^ carfilzomib. Data are represented as the geometric mean and 95%CI of the ratio of CLA relative to the pretreatment value

Carfilzomib specifically functions as an inhibitor of the chymotrypsin-like activity of the 20S proteasome, which leads to the accumulation of protein substrates within the cell and induction of apoptosis. We analyzed chymotrypsin-like activity in PBMCs in order to monitor the pharmacologic target of carfilzomib and to evaluate its correlation with efficacy in a preliminary fashion. Analysis of lysates from isolated PBMCs was conducted in 16 patients. Overall, there was a time dependent decrease in the chymotrypsin-like activity (CLA) of the proteasome. Figure 1 depicts the changes in CLA relative to pretreatment in the three dosing cohorts. Values are presented as the geometric mean and 95% CI. Decreased CLA activity was noted within two hours.

### Efficacy

There were three partial responses (PR), one per dose level, with one confirmed, for a overall (ORR) of 19%. One patient had stable disease (6%) for a disease control rate (DCR) of 25%. The confirmed PR occurred in a Cohort 1 patient (SCLC) who completed six cycles of treatment, and the response was sustained for four months following cessation of therapy. The two unconfirmed PRs occurred in a Cohort 2 patient who received four cycles and a Cohort 3 patient who received three cycles. Another Cohort 3 patient had stable disease after three treatment cycles. All cases of response and stable disease occurred in patients with SCLC. Two patients who withdrew voluntarily from treatment were not assessable for response. The remaining 12 patients had progressive disease. Two patients remained alive and progression free at analysis time. The median PFS estimate was 1.6 months (95% CI: 1.2–3.6 months). The estimated six month PFS rate was 7% (95% CI: 0%–20%).

## Discussion

The combination of irinotecan and carfilzomib was safe and tolerable with the MTD of 20/45 mg/m^2^ of carfilzomib and 125 mg/m2 of irinotecan in this patient population of irinotecan-sensitive solid tumors. Stepped-up dosing was incorporated into this trial based on observations of acute tumor lysis syndrome (TLS) in previous studies of carfilzomib in hematologic patients. However, no evidence of TLS was seen in the patients treated on this study. Premedication with dexamethasone led to a much lower rate of infusion reactions than reported in earlier studies; only one patient had a Gr 2 infusion reaction, which responded to medical management and did not recur after reinstitution of dexamethasone premedication. DLTs included fatigue, thrombocytopenia, and neutropenic fever. Other toxicities were manageable and included diarrhea, decreased white blood cells and neutrophils, and others listed in Table 3. Two subjects discontinued therapy because of poor tolerance, indicating that dose reduction and early management of diarrhea, dehydration, and low blood counts is important with this regimen to allow subjects to tolerate it.

Antitumor activity was noted in one subject with a PR after six cycles that was sustained for three additional months (SCLC) and two subjects with stable disease for greater than two cycles (SCLC and NSCLC). In this heavily pre-treated group of patients, the RR of 18% and DCR of 24% are encouraging, especially coupled one sustained PR for four months following completion of six cycles of therapy. The combination of carfilzomib and irinotecan is currently being evaluated in SCLC in the phase II portion of this study (NCT01941316).

Combination treatment using carfilzomib with irinotecan was anticipated to: a) enhance anti-tumor effect via inactivation of proteasome function and increased apoptosis; b) synergize with irinotecan via the different mechanisms of apoptosis of the compounds; and c) interfere with topo-I degradation, resulting in enhanced anti-tumor effect. Correlative studies confirmed decrease in chymotrypsin-like activity in the majority of patients, indicating proteasome inhibition was potent, ongoing, and not adversely affected by irinotecan.

In summary, the combination of irinotecan and carfilzomib was well tolerated in this heavily pretreated cohort of subjects with relapsed solid tumors. The most common toxicities were fatigue, thrombocytopenia and neutropenic fever. Objective clinical response was seen in one patient with SCLC. A dose of carfilzomib 20/36 mg/m^2^ and irinotecan125 mg/m2 was selected for further evaluation in the ongoing phase II study of this combination in SCLC. This phase II evaluation will help further characterize the efficacy of this combination in relapsed SCLC.

## Abbreviations

AE: Adverse Event
ALT: Alanine Transaminase
AST: Aspartate Aminotransferase
BSA: Body Surface Area
CLA: Chymotrypsin-Like Activity
CTCAE: Common Terminology Criteria For Adverse Events
BSA: Body Surface Area
DCR: Disease Control Rate
DLT: Dose Limiting Toxicity
FDA: Food And Drug Adminstration
Gr: Grade
HIV: Human Immunodeficiency Virus
IRB: Institutional Review Board
i.v.: Intravenous
MTD: Maximum Tolerated Dose
NYHA: New York Heart Association
NSCLC: Non-Small Cell Lung Cancer
NF-κB: Nuclear Factor-κB
PR: Partial Responses
PBMC: Peripheral Blood Mononuclear Cell
PFS: Progression-Free Survival
RECIST: Response Evaluation Criteria In Solid Tumors
RR: Response Rate
SCLC: Small Cell Lung Cancer
TLS: Tumor Lysis Syndrome
